# Overview of the Americas’ First Peopling from a Patrilineal Perspective: New Evidence from the Southern Continent

**DOI:** 10.3390/genes13020220

**Published:** 2022-01-25

**Authors:** Giulia Colombo, Luca Traverso, Lucia Mazzocchi, Viola Grugni, Nicola Rambaldi Migliore, Marco Rosario Capodiferro, Gianluca Lombardo, Rodrigo Flores, Monika Karmin, Siiri Rootsi, Luca Ferretti, Anna Olivieri, Antonio Torroni, Rui Martiniano, Alessandro Achilli, Alessandro Raveane, Ornella Semino

**Affiliations:** 1 Department of Biology and Biotechnology “L. Spallanzani”, University of Pavia, 27100 Pavia, Italy; giulia.colombo01@universitadipavia.it (G.C.); or luca_traverso@eva.mpg.de (L.T.); lucia.mazzocchi01@universitadipavia.it (L.M.); viola.grugni@unipv.it (V.G.); nicola.rambaldi01@universitadipavia.it (N.R.M.); marcorosario.capodiferro01@universitadipavia.it (M.R.C.); gianluca.lombardo01@universitadipavia.it (G.L.); luca.ferretti@unipv.it (L.F.); anna.olivieri@unipv.it (A.O.); antonio.torroni@unipv.it (A.T.); alessandro.achilli@unipv.it (A.A.); 2 Present address: Department of Archaeogenetics, Max Planck Institute for Evolutionary Anthropology, Deutscher Platz 6, 04103 Leipzig, Germany; 3 Estonian Biocentre, Institute of Genomics, University of Tartu, 51010 Tartu, Estonia; rodrigo.flores@ut.ee (R.F.); monika.karmin@ut.ee (M.K.); siiri.rootsi@ut.ee (S.R.); 4 School of Biological and Environmental Sciences, Liverpool John Moores University, Liverpool L3 3AF, UK; R.D.LeitePortelaMartiniano@ljmu.ac.uk

**Keywords:** Y-chromosome variation, peopling of the Americas, haplogroups C and Q, phylogeny, inland migrations

## Abstract

Uniparental genetic systems are unique sex indicators and complement the study of autosomal diversity by providing landmarks of human migrations that repeatedly shaped the structure of extant populations. Our knowledge of the variation of the male-specific region of the Y chromosome in Native Americans is still rather scarce and scattered, but by merging sequence information from modern and ancient individuals, we here provide a comprehensive and updated phylogeny of the distinctive Native American branches of haplogroups C and Q. Our analyses confirm C-MPB373, C-P39, Q-Z780, Q-M848, and Q-Y4276 as the main founding haplogroups and identify traces of unsuccessful (pre-Q-F1096) or extinct (C-L1373*, Q-YP4010*) Y-chromosome lineages, indicating that haplogroup diversity of the founder populations that first entered the Americas was greater than that observed in the Indigenous component of modern populations. In addition, through a diachronic and phylogeographic dissection of newly identified Q-M848 branches, we provide the first Y-chromosome insights into the early peopling of the South American hinterland (Q-BY104773 and Q-BY15730) and on overlying inland migrations (Q-BY139813).

## 1. Introduction

The Americas were the last continents to be colonised by modern humans. For decades, scientists of different disciplines have debated the modes and times of their peopling [1,2,3,4,5] reaching a consensus on the Asian origin of the first settlers, their entrance from Siberia across Beringia before 16 thousand years ago (kya) or even earlier [6,7], and a rapid southward migration to the Southern Cone as attested by Monte Verde in Chile (~14.5 kya) and other archaeological sites [8,9]. In the last decade, genomic analyses of modern and ancient individuals have identified at least two ancestral Pleistocene components that, once entered into North America, underwent splits and admixture while moving southward [10,11,12]. To explain these results, different models have been proposed [13]. More recently, additional Pleistocene Indigenous components carried by still unsampled ancient populations (UPopA, UPopI) were identified in Central and South America [11,14,15], adding further complexity to the peopling scenarios. One of the main obstacles in obtaining a detailed genetic picture of the Americas first peopling is the lack of clear and complete information about the autochthonous gene pool because of the European colonization, the subsequent slave trade, and numerous more recent events of gene flow. Due to the uneven male/female native population decline and the high historical rate of European male-mediated admixture into Native American communities, the Y chromosome gene pool was the most affected [16,17,18,19].

Although autosomal genome-wide data allow researchers to investigate genetic admixture [20,21], and autosomes vs. X chromosome comparisons can assess sex-biased genetic admixture [22,23,24,25,26,27], only the uniparentally transmitted genetic systems, the maternally inherited mitochondrial DNA (mtDNA), and the male-specific region of Y chromosome (MSY) provide direct insights into the paternal and maternal sources of genetic ancestry. In addition, the fine-calibrated mtDNA and MSY molecular clocks [28,29,30] are key elements to date prehistoric and historical events.

Although mtDNA founding haplogroups have already been well-characterised in the literature [31,32,33,34], Native American MSY lineages still lack a high level of phylogenetic resolution. This is mainly due to the heterogeneity of available data, consisting of markers obtained either by sequencing targeted informative Y-chromosome regions [35,36,37,38], by extracting Y-chromosome data from whole genome shotgun sequencing [15,29,39,40,41], or through enrichment of selected sequences from ancient remains [14,42,43].

Here, we gathered Native American MSY sequence data from different approaches and sources, which were analysed through a novel workflow. The aim was to obtain a comprehensive and ascertainment-bias-free phylogeny as well as a diachronic view of continental Indigenous MSY variation.

## 2. Materials and Methods

### 2.1. Dataset Construction

Our dataset was obtained by retrieving modern and ancient Y-chromosome sequences potentially belonging to Native American haplogroups C and Q from the literature. In addition, worldwide samples from the Human Genome Diversity Project [41] representative of the major Y-chromosome haplogroups (A, B, C, E, I, G, J, O, R, and T) were included in the phylogenetic analysis. All assessed samples are listed in Table S1, with additional details, including the type of analysis performed.

The modern dataset consists of 425 males with Y chromosomes clustering within haplogroups C or Q [10,29,36,37,38,39,40,41,44,45]. Sequences were divided into High Coverage (HC, *N* = 147) and Low Coverage (LC, *N* = 278). The ancient dataset includes 293 samples belonging to haplogroups C and Q [11,12,14,42,43,46,47,48,49,50,51,52,53,54,55]. The American double continent is represented by 370 modern and 262 ancient subjects whose geographic locations are shown in Figure S1. The phylogeography of the most common C and Q sub-haplogroups was evaluated by also considering samples for which only the SNP genotyping classification was available (Table S1).

### 2.2. Filtering and Coverage Estimation

Given the heterogeneity of the dataset, sequences were converted to the BAM format, unless only VCF files were available. The BWA-MEM algorithm v. 0.7.17 [56] was used to align FASTQ sequences to the Hg19 human genome reference, and the SAMtools view option [56] was employed to extract the Y chromosome from whole genome data. 

Coverage estimation was assessed using the SAMtools depth command. Furthermore, we calculated the percentage of positions covered in the total of ~10 Mb regions of interest defined by Poznik et al. [35], by using a minimum depth threshold equal to 1. High Coverage and Low Coverage samples were defined as those with a covered percentage of the region of interest higher or lower than 66%, respectively. Samples that were only available in a VCF format were included in the HC category based on the filters applied in the original studies [10,29].

### 2.3. Variant Calling, Merging, and Quality Control Filters

FreeBayes 1.3.4 [57] was used to perform variant calling within the ~10 Mb regions defined by Poznik et al. [35], applying the following options: minimum mapping quality = 20, minimum base quality = 20, minimum depth of coverage = 3, and non-ALT base exclusion (--report-monomorphic); all the other settings were left as default. HC samples were merged into a single VCF, excluding non-variant positions, InDels, Multi Nucleotide Polymorphism (MNPs), as well as positions present in less than 90% of samples. Y-chromosome haplogroup assessment of each HC sample was performed with Y-LineageTracker [58], and pivotal markers, coherent with the haplogroup classification, were imputed into the VCF file. This was performed in order to minimise the bias caused by the heterogeneity of sequenced regions.

Finally, the multi-VCF was converted into PHYLIP format using the python script (vcf2phylip.py) introduced by Ortiz [59].

### 2.4. Phylogenetic Analysis

RAxML v.8.2 (Randomized Axelerated Maximum Likelihood) [60] was used to perform the phylogenetic analysis, by running 1000 bootstrap replicates with substitution model ‘‘ASC_GTRGAMMA’’ as in Pinotti et al. [38] and with ascertainment-bias correction model “lewis”. A python script (Afterphylo.pl, https://github.com/qiyunzhu/AfterPhylo, accessed on 3 December 2021) was employed to cut off from the final tree all the branches that were drawn in less than 10% of bootstrap repetitions. Finally, the tree was re-rooted with the Ete3 python package [61] setting the sample HGDP01029 (haplogroup A1) as outgroup [41].

Phynder software (https://github.com/richarddurbin/phynder, accessed on 3 December 2021) was used to assign SNPs to the re-rooted tree, and pathPhynder [62] allowed the classification of LC and ancient samples (AS). Finally, the output classification was evaluated and manually adjusted for inconsistencies.

### 2.5. Time Estimates of Phylogenetic Nodes

The effective population size analysis was estimated on consensus multi-FASTA files, produced from the combined multi-VCF dataset (assembled as described above) using a reference-free python script created for this purpose, based on scikit-allel [63]. Molecular dating of the main haplogroup Q sub-branches was then performed with a single BEAST v2.6.6 analysis [64] using all HC samples. The following parameters were applied: coalescent Bayesian skyline, a GTR substitution model (most fitting substitution model, both in terms of BIC and AICc, as defined by “Find best DNA/Protein Models” MEGAX function [65]), substitution rate of 1.74 × 10^−10^ [29], a relaxed clock rate, the median calibrated dates of ancient DNAs as calibration points, 50 million iterations, sampled every 1000 steps. The results obtained were combined after discarding the first 20% of each replicate as burn-in using LogCombiner v.1.8.3 (http://beast.community/logcombiner, accessed on 3 December 2021). The trees were summarised using TreeAnnotator v.1.8.3 (http://beast.community/treeannotator, accessed on 3 December 2021). The highest posterior densities (HPD), which collect the most likely age distributions, were calculated for each TMRCA considered, taking into account the Effective Sample Size (ESS) parameter. Coalescent time estimates were obtained by combining .log files after discarding the first 2000 generations of each replicate as burn-in, and the results were visualised in Tracer v1.7 [66]. Bayesian skyline plots (BSPs) were generated with Tracer v1.7 using as input the files obtained in the previous BEAST analyses, recalibrated on the onset of a new generation every 25 years and plotted with R [67].

## 3. Results

**Figure 1:**
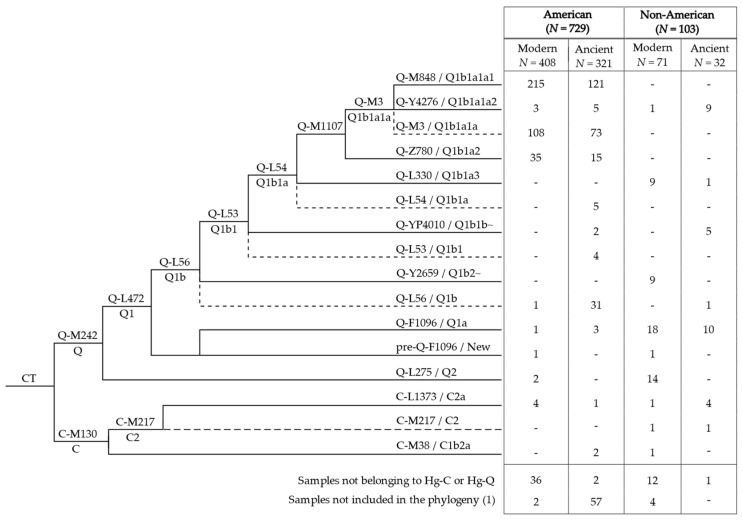
Structure of the main haplogroup C and Q lineages observed in our dataset. In bold are reported the absolute number (*N*) of American and non-American individuals analysed. Dashed lines harbour samples classified into the related haplogroup but not better defined (missing positions of informative downstream markers). ISOGG nomenclature (http://www.isogg.org/tree/ Date of access: 19 January 2022) is reported, when available; “New” indicate branches not reported. The table reports the absolute number of individuals (*N*) belonging to each haplogroup. (1) Samples not placed in the phylogeny due to their poor sequencing quality.

Out of the 832 assessed Y chromosomes, 15 fell into haplogroup C and 703 into haplogroup Q (Figure 1 and Figure S2). The remaining 114 sequences were excluded from further analyses, as they were classified into non-American haplogroups (*N* = 51), or they were too poor in quality to be placed in the phylogeny (*N* = 63).

### 3.1. Phylogeny and Phylogeography of Haplogroup C Y Chromosomes

Haplogroup C is a non-African haplogroup observed in Eurasia and one of the two main clades characterising Native American Y chromosomes. It has been observed at low frequencies in Athabascans and Algonquians from North America and in Indigenous groups from north-western South America [17,68,69,70,71,72,73]. The North American Y chromosomes were characterised by the marker P39 [69], whereas those from South America, initially indicated as C3*, were recently classified as C-MPB373 [38], a sub-lineage of C-L1373, which is mainly present in North Eurasia.

**Figure 2:**
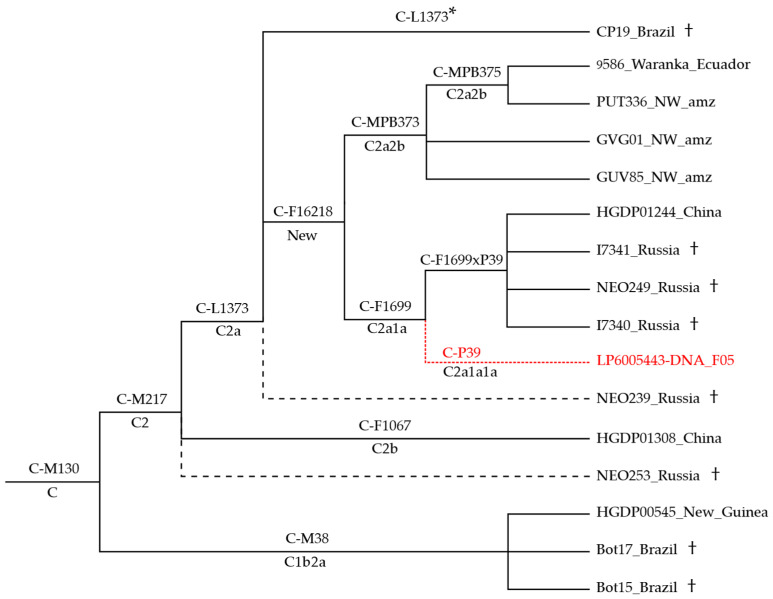
Phylogeny of haplogroup C sub-branches. C-L1373* identifies a Brazilian sample negative for the markers of all other C-L1373 derived branches; dashed black lines indicate less-well-defined samples (missing positions of informative downstream markers); (†) marks ancient samples. ISOGG nomenclature (http://www.isogg.org/tree/, accessed on 19 January 2022) is reported, when available; “New” indicate branches not reported. The placement of the North American C-P39 (in red) is inferred from Pinotti et al. [38].

The phylogenetic structure of haplogroup C highlights the lack of American Hg C Y chromosomes that could be useful to further resolve the phylogeny (Figure 2). Out of the seven American Hg C samples included in our dataset, two ancient Brazilians (Bot15 and Bot17; ~500 years ago) belong to C-M38, a sub-haplogroup frequent in Indonesia and Oceania [74]. Their presence in South America has been attributed to colonial exchanges [49]. The remaining are four modern samples from Colombia (PUT336, GUV85, and GVG01) and Ecuador (9586_Waranka) and one ancient Brazilian specimen (CP19 from Lapa do Santo; 9.85 kya). All of them fall into the recently described sub-clade C-L1373 [38], together with a few far-North-East Asians (Figure S3). In particular, the three Colombian and the Ecuadorian cluster into C-MPB373, suggesting that this branch might be specific to north-western South America.

Due to public raw data unavailability, our dataset does not include sequences belonging to C-P39, a branch that stems from C-F1699 according to Wei et al. [75] and Pinotti et al. [38], parallel to C-F1699(xP39) that encompasses samples (ancient and modern) from Siberia and China.

Interestingly, the ancient Brazilian sample CP19 fell into C-L1373 but carried ancestral alleles for C--MPB373, C-F1699, and downstream markers, including B473 that characterises the North American C-P39 branch [38]. Therefore, the ancient specimen was classified as C-L1373* identifying C-F16218, a new parallel branch comprising all the above-noted lineages.

### 3.2. Phylogeny and Phylogeography of Haplogroup Q Y Chromosomes

Haplogroup Q chromosomes are currently found in Eurasia and the Americas [76], where they are almost entirely represented by the Q-M1107 clade [37,38]. Out of the 703 samples that we classified as members of haplogroup Q, 625 were from America and only 9 of them belonged to lineages that are not American-specific: Q-L275, Q-F1096, and Q-YP4010.

Q-L275 characterised two Central American samples (NA56 from Panama and AMZ139 from Colombia) belonging to its sub-branch Q-L245, which was previously reported across Eurasia [30].

Q-F1096 was represented by Q-M120 (frequent in South-East Asia) in the Peruvian HG01944, and by Q-B143 (found in Siberia and North America) in ancient samples from Alaska (I1126; 1.2 kya) and Greenland (Saqqaq; 3.8 kya). Moreover, a 1.9 ky-old sample from Canada (I10427) was also classified as Q-F1096 but could not be better defined due to the lack of informative markers for downstream haplogroups. Interestingly, a modern sample from Alaska (Tsimshian) branches off prior to the Q-F1096 node, sharing only two (out of 30) of its distinguishing mutations. 

Finally, haplogroup Q-YP4010 (present across Asia), in America was only found in two ancient samples: a 1.8 ky-old from Lovelock Cave, Nevada (Lovelock4) and a 500-year-old Paleo-Aleut from the Aleutian Islands (I0719). Both were classified outside a cluster of ancient Siberians dated between 7 and 4.6 kya. 

**Figure 3:**
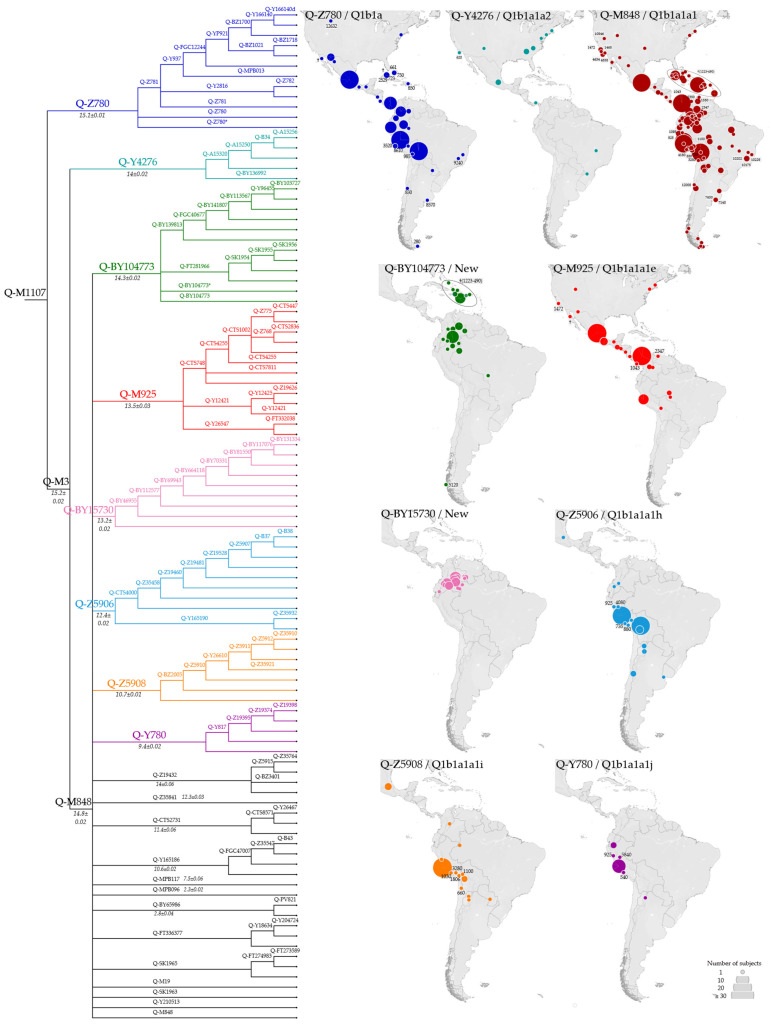
Phylogenetic structure of the American-specific haplogroup Q-M1107. The maps on the right show the geographic distribution of the major Q-M1107 sub-lineages. Ancient individuals are indicated with their median calibrated age (cal BP, see Table S1 for details), or with a cross when age was not available. ISOGG nomenclature (http://www.isogg.org/tree/, accessed on 19 January 2022) is reported, when available; “New” indicate branches not reported. In the phylogeny, the estimated ages of the main node (±StDev) are reported in kya.

The vast majority of the American samples (575 individuals of the 670 placed in the phylogeny, Figure 1) belonged to Q-M1107. This haplogroup includes Q-Z780 and Q-M3, both previously described as pan-American founders [37,38], as well as Q-L804, observed at very low frequency in Northern Europe [75,77] and not represented in the dataset used to build our phylogeny. Haplogroups Q-Z780 and Q-M3 and their main branches (Figure 3 and Figure S2) are present in different proportions throughout the Americas. In particular, Q-Z780 was observed at a lower incidence (50 over 670) compared to Q-M3 (525 over 670), which was confirmed as the most frequent pan-American haplogroup (Figure 1). Within haplogroup Q-M3, the sub-lineage Q-Y4276 was observed at relatively low frequency and distributed mainly in North America, whereas Q-M848 characterised the great majority of American Y chromosomes.

**Figure 4:**
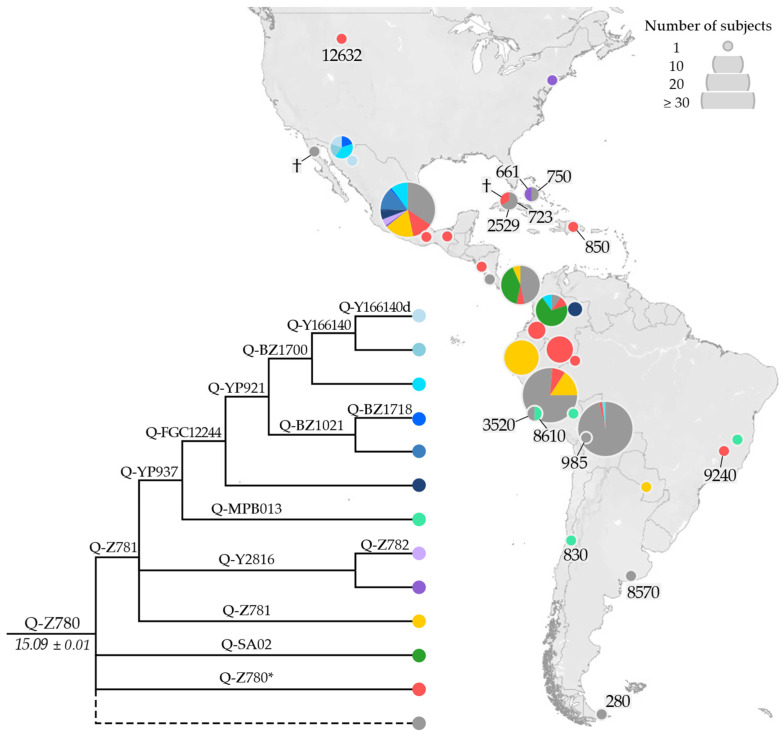
Geographical distribution of Q-Z780 (ISOGG nomenclature: Q1b1a2—http://www.isogg.org/tree/, accessed on 19 January 2022) and its main sub-lineages. Ancient individuals are indicated with their median calibrated age (cal BP, see Table S1 for details), or with a cross when the age was not available. The dashed line comprises Q-Z780 samples less well defined (missing positions of informative downstream markers). In the phylogeny, the estimated age of the node (±StDev) is reported in kya.

The phylogenetic structure of haplogroup Q-Z780 (Figure 4) was substantially enriched in comparison with previous studies [37,38]. Its single well-characterised sub-branch, Q-Z781, encompasses the minor Q-Y2816 and the major Q-YP937. Whereas Q-Y2816 is almost exclusively found in Mexico, the Q-YP937 sub-lineages, Q-MPB013 and Q-FGC12244, occupy different areas.

Q-MPB013 was only detected in South America in an 8.6 ky-old ancient specimen from Peru (I0038), a 0.8 ky-old ancient sample from Chile (I1754), and two modern individuals from Peru (9597_Aymara) and Brazil (9585_Maxacali). Moreover, Q-FGC12244, the other Q-YP937 sub-lineage, was only observed from Mesoamerica to the Isthmo-Colombian area. The structure of this sub-branch was noticeably improved in comparison with previous studies, with the identification of Q-BZ1700 and its sub-lineages Q-Y166140 and Q-Y166140d, which comprise almost exclusively Mexican samples. 

It is of note that the previously reported Q-SA02 [37,78], parallel to Q-Z781, is not represented in our phylogenetic analysis (Figure 3) due to the lack of HC data. Therefore, two Panamanian LC samples (NA5 and NA62), classified as Q-SA02 by [37], are here classified as Q-Z780 (xZ781). This minor lineage appears to be restricted to the Isthmo-Colombian area.

Overall, although Q-Z780 was observed throughout the double continent, its frequency remains consistently inferior compared to Q-M3. In fact, Q-M848, the most represented Q-M3 sub-lineage, is by far the most frequent and widespread haplogroup throughout the Americas [37,38].

In this work, 18 Q-M848 sub-lineages were observed, six reported here for the first time. Most of Q-M848 sub-lineages include few samples, but some of them (Q-M925, Q-Z5906, Q-Z5908, Q-Y780, and the new Q-BY104773 and Q-BY15730) appear well-structured and locally differentiated, with no major signs of propagation in neighbouring areas.

**Figure 5:**
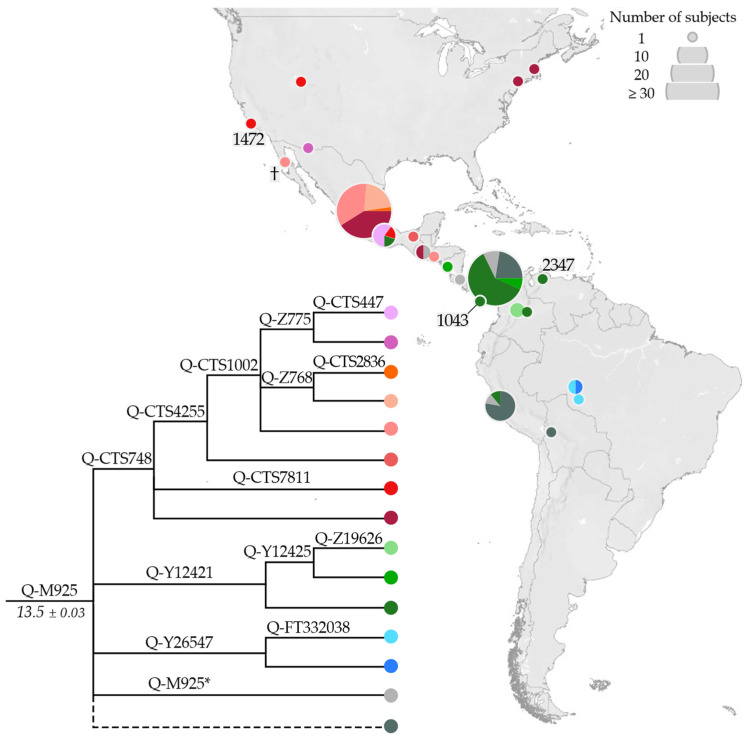
Geographical distribution of Q-M925 (ISOGG nomenclature: Q1b1a1a1e—http://www.isogg.org/tree/, accessed on 19 January 2022) and its main sub-lineages. Ancient individuals are indicated with their median calibrated age (cal BP, see Table S1 for details), or with a cross when the age was not available. The dashed line indicates Q-M925 samples less well defined (missing positions of informative downstream markers). In the phylogeny, the estimated age of the node (±StDev) is reported in kya.

Q-M925 (Figure 5) is most frequent in Mesoamerica and the Isthmo-Colombian area; it includes three branches (Q-CTS748, Q-Y12421, and Q-Y26547) with different geographic distributions: Q-CTS748 is mainly observed in Mexico and not reported south of El Salvador; Q-Y12421 reaches considerable frequencies in the Isthmo-Colombian area [19]; Q-Y26547 is only found in three Brazilians from Amazonia [41]. Although not represented by any sequence, Q-M925* was reported at a moderate frequency in Panama [19] and in a few samples from Costa Rica, Guatemala, and Peru [37,79]. Our phylogeographic results (Figure 5) are in accordance with previous reports. Moreover, the addition of ancient samples (the Meso-American PS_07 and B_3 in Q-CTS748, the Venezuelan I17889, and the Panamanian PAPV117 in Q-Y12421) highlights a territorial continuity of this lineage.

**Figure 6:**
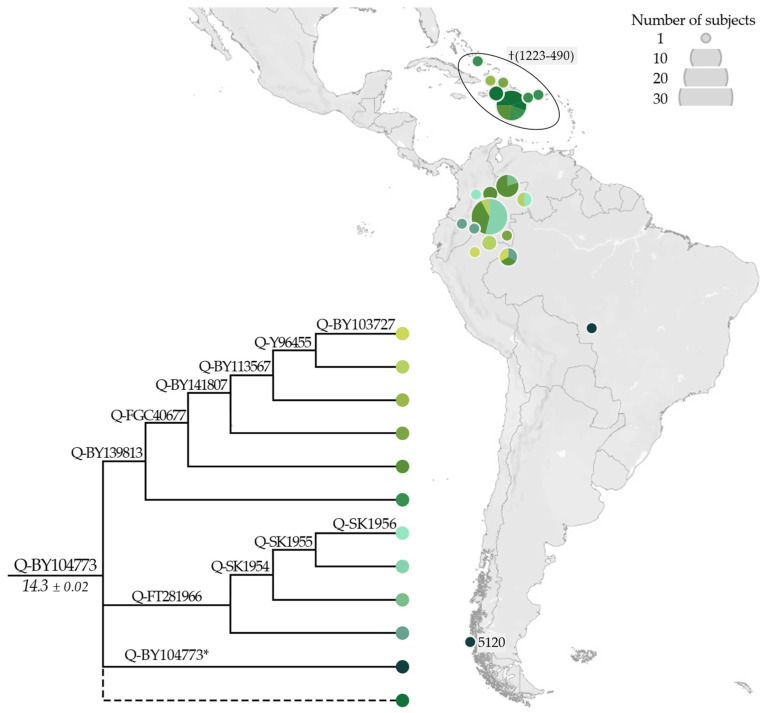
Geographical distribution of Q-BY104773 (not reported in ISOGG, http://www.isogg.org/tree/, accessed on 19 January 2022) and its main sub-lineages. Ancient individuals are indicated by their median calibrated age (cal BP, see Table S1 for details), or with a cross when the age was not available. The dashed line indicates Q-BY104773 samples less well defined (missing positions of informative downstream markers). In the phylogeny, the estimated age of the node (±StDev) is reported in kya.

The newly identified Q-BY104773 lineage (Figure 6) includes two branches, Q-BY139813 and Q-FT281966. Both sub-lineages characterise modern individuals from North-West Amazonia, but only Q-BY139813 also includes a group of ancient (~1.3–0.5 kya) Y chromosomes from the southern Caribbean area. A single ancient sample from Ayayema Cave in Chile (A460; 5.1 kya) and a modern Brazilian (9595_Nambikwara, previously reported as Q-M848 [38]), were classified as Q-BY104773*, as they did not belong to either of the two branches.

**Figure 7:**
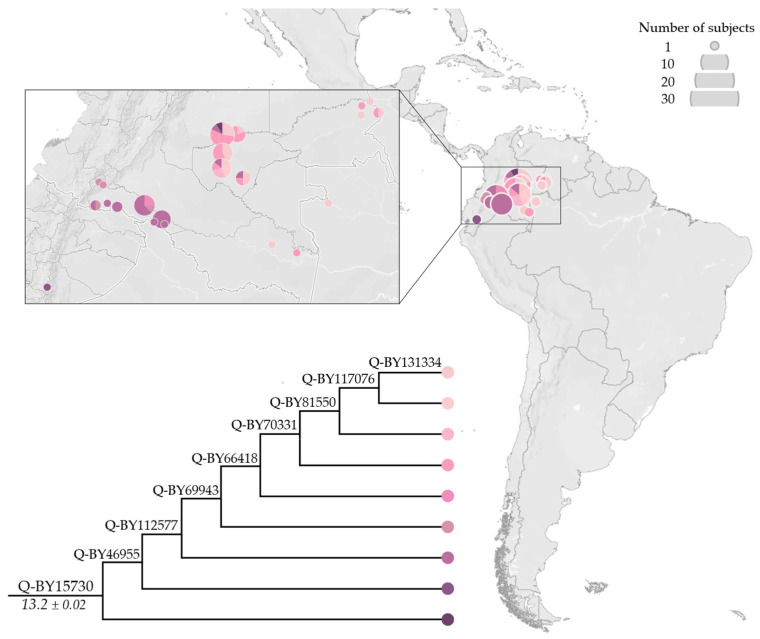
Geographical distribution of Q-BY15730 (not reported in ISOGG, http://www.isogg.org/tree/, accessed on 19 January 2022) and its main sub-lineages. In the phylogeny, the estimated age of the node (±StDev) is reported in kya.

The newly characterised Q-BY15730 (Figure 7) partially overlaps the distribution range of Q-BY104773, and encompasses modern-day individuals from North-West Amazonia, mostly from Tukanoan-speaking groups [36]. Eight sub-lineages were identified, showing a gradient-like distribution from the Ecuadorian Andes to the border between Colombia and Venezuela, with more basal sub-lineages in the West and younger-nested sub-lineages in the East.

**Figure 8:**
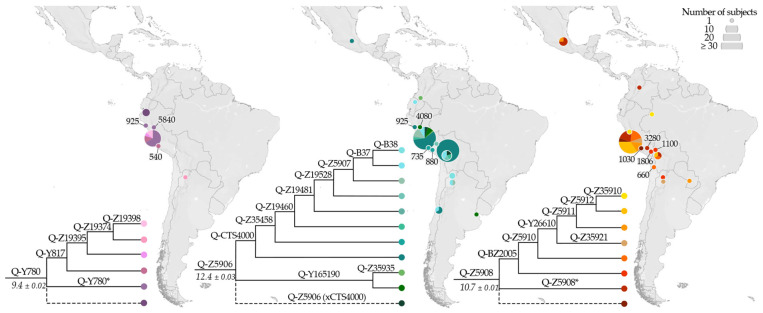
Geographical distribution of Q-Y780, Q-Z5906, and Q-Z5908 (ISOGG nomenclature: Q1b1a1a1j, Q1b1a1a1h, and Q1b1a1a1i, respectively—http://www.isogg.org/tree/, accessed on 19 January 2022) and their main sub-lineages. Ancient individuals are indicated with their median calibrated age (cal BP, see Table S1 for details). Dashed lines indicate samples less well defined (missing positions of informative downstream markers). In the phylogeny, the estimated ages of the nodes (±StDev) are reported in kya. (*) identifies samples negative for the markers of all derived branches.

The remaining geographically well-structured Q-M848 lineages are distributed along the Pacific Coast: the minor Q-Y780 sub-haplogroup is confined to the Andean territory, whereas Q-Z5906 and Q-Z5908 include a fairly small number of individuals (2 out 122, 6 out 73, respectively) on the other side of the mountain range (Figure 8). 

For Q-Y780 a single sub-lineage, Q-Y817, was identified leaving nearly half of the Q-Y780 sequences classified as Q-Y780*, including a 5.84 ky-old Peruvian sample.

In contrast, Q-Z5906 and Q-Z5908 are more frequent and more structured compared to Q-Y780 and are highly frequent in Peru. Notably, Q-CTS4000, a major sub-branch of Q-Z5906, was expanded with the identification of seven sub-lineages that comprise modern and ancient Andean samples from Ecuador to Chile and an isolated Mexican individual classified as Q-CTS4000 (xB37) as previously observed in [37]. Parallel to Q-CTS4000, the minor sub-clade Q-Y165190 represents a few samples from the Andes, including a 4.08 ky-old sample from Chile (I2260) and a modern individual from Argentina.

The distribution of Q-Z5908 resembles that of Q-Z5906 and it is mainly made up of present-day Andean individuals. However, unlike Q-Z5906, Q-Z5908 includes more samples out of the mountain range and a larger number of ancient individuals. In particular, the 3.28 ky-old CUN008 from Peru defines a new sub-lineage, Q-BZ2005, upstream of the previously identified Q-Z5910 [37,38]. Q-BZ2005 harbours two ancient samples from Bolivia (MIS7) and Peru (Lake Titicaca, IL7) both dated between 2 and 1 kya, together with a modern Argentinian.

Finally, other smaller and geographically poorly structured clusters were identified downstream of Q-M848, namely: Q-Z19432, Q-Y165186, Q-FT336377, Q-CTS2731, Q-SK1965, Q-BY65986, Q-M19, Q-Z35840, Q-SK1963, Q-Y210513, Q-MPB117, and Q-MPB096 (Figure 3 and Figure S2). Notably, most of these sub-clades, although quite rare, are unevenly scattered throughout South America (Figure S4). This suggests that they represent what is left of unsuccessful lineages that originated before spreading throughout the subcontinent, rather than the legacy of multiple recent locally restricted events. However, the small number of encompassed samples limits the possibility of drawing clear-cut conclusions on their origin and dispersal.

### 3.3. Age and Population Growth Estimates

**Figure 9:**
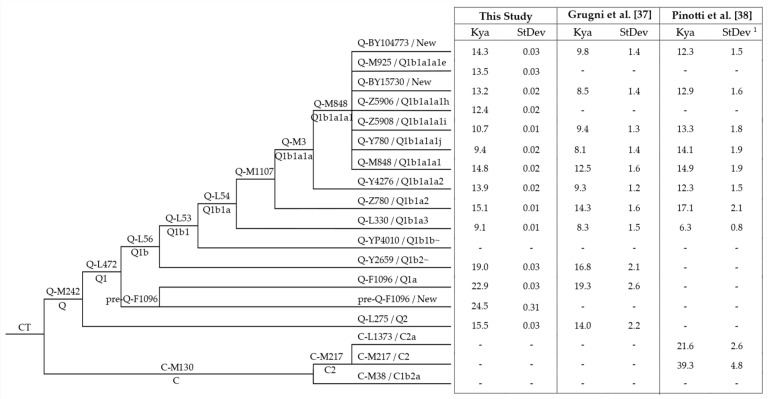
Age estimates of the haplogroup C and Q branches in comparison with those from previous studies (more details in Table S2). ISOGG nomenclature (http://www.isogg.org/tree/, accessed on 19 January 2022) is reported, when available; “New” indicate branches not reported. ^1^ Standard deviation for the age estimate in Pinotti et al. [38] was calculated from upper and lower bounds.

Ages were estimated for all the main haplogroups of the phylogeny and, when the number of represented samples allowed it, for their sub-haplogroups (Table S2). In addition, we compared the obtained estimates of the major clades with those previously reported [37,38] (Figure 9).

**Figure 10:**
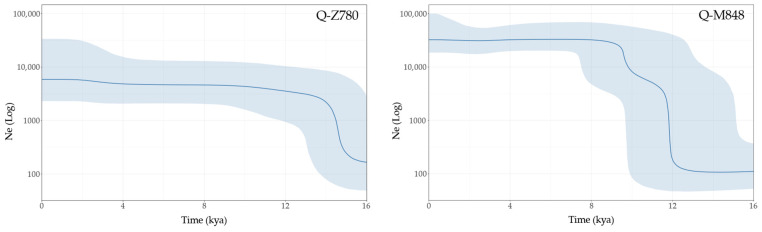
Bayesian skyline plots of Q-Z780 and Q-M848. Only Native American high coverage (HC) Y chromosomes were considered (*N* = 13, *N* = 81, respectively). The radiocarbon dates of the following ancient individuals were used as priors for time estimates: Anzick-I [47], Ahur, Sumidouro5, A460, [11], CUN008 [14] for haplogroup Q-M848; I2261 and I0038 [14] for Q-Z780. The x-axis is in thousand years ago, and the y-axis shows changes in effective population size in logarithmic scale. The darker lines trace the median estimates, and the shadings show 95% highest posterior density intervals of the Ne. The time axis is limited to 16 kya, beyond which the curve remains flat.

Finally, the effective population size estimates of Q-Z780 and Q-M848 are shown in Figure 10.

The effective population size of Q-Z780 rapidly increases between 15 and 14 kya, slowly reaching a plateau around 10 kya, until a second, slight sign of increase is observed at 4 kya. In contrast, Q-M848 shows two rapid population growths, at 12.5 kya and at 9 kya, occurring in less than one millennium and separated by a very low, but steady, three millennia expansion.

**Figure 11:**
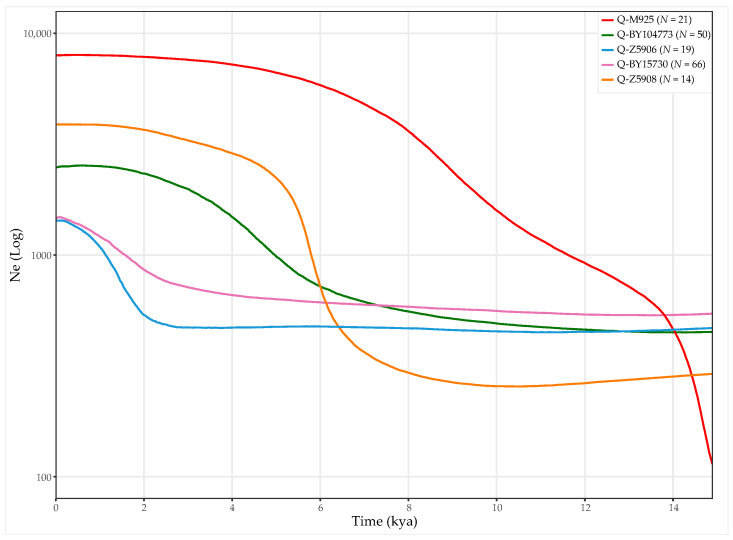
Bayesian skyline plots of the most represented Q-M848 sub-clades. All Native American (HC and LC) Y chromosomes were considered: the number of individuals per sub-lineage is reported in the inset. Timing of events was estimated based on all the available ancient sample’s radiocarbon dates (Table S1). The x-axis is in thousand years ago, and the y-axis shows changes in effective population size in logarithmic scale. The coloured lines trace the median estimates of the related clade. The time axis is limited to 15 kya, beyond which the curve remains flat.

From the analysis carried out in the major Q-M848 sub-clades, it appears that the rapid growths observed for Q-M848 are mainly due to the Q-M925 branch (Figure 11), which masks the feeble increment of population size registered after 5 kya for the other lineages.

## 4. Discussion

For decades, scientists have tried to shed light upon America’s first peopling and the time and modes of the subsequent dispersal events. One of the main hurdles in disentangling the genetic history of the American populations is the lack of detailed information on their gene pool. This is due to the impact of European colonisation and the subsequent slave trade. These events, associated with wars and diseases, led to a dramatic reduction of the Indigenous people and therefore of their contribution to the present-day American gene pool. This decrease was particularly accentuated for the Y chromosome, due to the uneven male/female Native population decline and the high historical rate of male-mediated admixture into Native American communities [27,37,80,81,82]. Now, archaeogenomics fills this gap, allowing us to compare ancient with modern genomes [83], although merging all types of Y-chromosome data is complicated by the heterogeneity of markers analysed and regions investigated [62].

In this work, Y-chromosome data from several studies were employed to construct the most up-to-date phylogeny of the Native American founding lineages. A dataset of the Y-chromosome sequences potentially ascribable to American Indigenous clades was assembled and a computational workflow was developed to insert modern LC and ancient samples into a robust reference phylogeny built only with HC sequences. This approach, based on the imputation of missing data in LC and ancient samples, allowed us to minimise the bias caused by the different methods used in the original studies [62,84].

**Figure 12:**
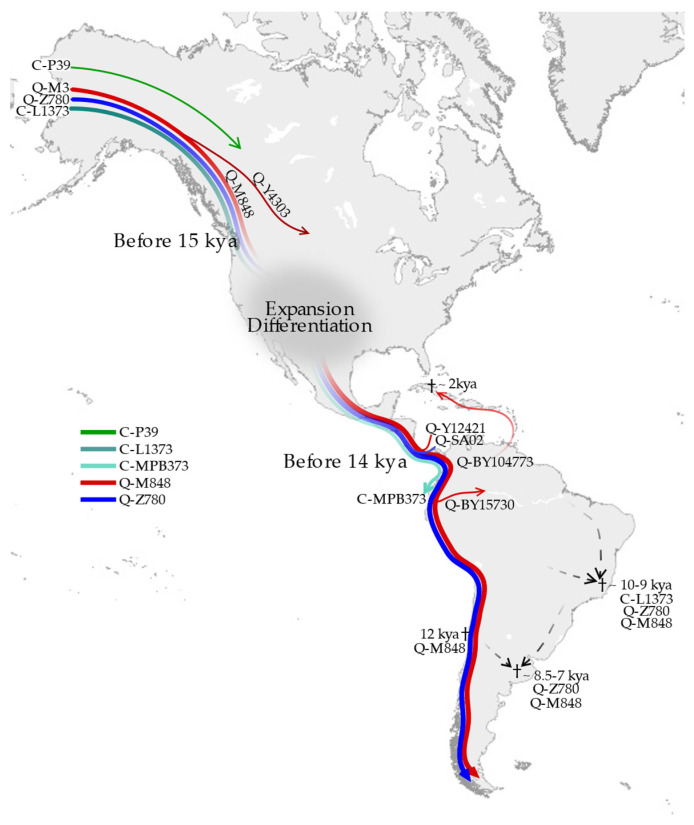
Graphical summary of the main migratory events from North towards South America according to the Y-chromosome variation. Dashed lines represent hypothetical migratory routes; (†) marks archaeological sites.

The phylogeny that we obtained supports and details the structure of Native American haplogroups. We significantly increase the resolution of Q-M1107 and confirm haplogroups C-MPB373, Q-Z780, Q-M848, and Q-Y4276, together with C-P39 (not represented in our phylogeny dataset), as the main Native American founding lineages. The paragroup C-L1373*, observed only in one ancient sample, is a novel enrichment of the haplogroup C phylogeny. The phylogeography of the major branches of the above-noted haplogroups indicates that C-L1373*, C-MPB373, Q-Z780, and Q-M848 were the first to colonize the Americas, rapidly reaching the Southern Cone (Figure 12). However, the present-day distribution of these clades as well the location and age of the affiliated ancient samples indicate that their diffusion was due to different demographic events.

First of all, haplogroup C, beside the North American C-P39, which entered the continent with subsequent migrations [38], is only represented by a 9.85 ky-old C-L1373* sample (CP19) from Lapa do Santo and by the C-MPB373 cluster. This sub-haplogroup is placed downstream of C-L1373; it is mainly represented by present-day north-western Amazonians, and a rough age estimate based mainly on LC data (only one sequence was HC) suggests an ancient origin (9.4 + 0.1 kya). Thus, whereas the ancient Brazilian CP19 highlights the presence of C-L1373 in South America since at least 10 kya, the observation of a different sub-clade in South-West America supports the scenario of two C-L1373 lineages arriving with the first settlers. These lineages followed different routes and were probably underrepresented, as one did not yield modern descendants and the other is now restricted to very isolated Indigenous groups.

Q-Y4276, the minor clade of haplogroup Q-M3, is mainly observed in North America and only sporadically in Siberia, Panama, and Brazil. Its age (13.9 kya) confirms an early entrance, whereas its presence in Siberia has been attributed to a recent back migration [37]. No information is available to understand if the few subjects harbouring Q-Y4276 in Panama and Brazil are the legacy of ancient migrations or the outcome of recent movements.

Unlike Q-Y4276, Q-Z780 and Q-M848 are observed throughout the Americas. Q-Z780 is the most ancient clade (15.1 kya) and has been found in ancient samples across the whole double continent—from Montana (Anzick-I, 12.6 kya) to Peru, Brazil, and Argentina (9.2–8.6 kya). Yet, in modern individuals, Q-Z780 is mainly observed in Mexico as Q-Z781. In contrast, Q-M848, which is slightly younger than Q-Z780 (14.8 kya), is much more frequent and structured, including many parallel sub-clades, each with a specific geographic localisation, suggesting several early differentiation events and a rapid southward expansion.

Q-M925, occupying the northernmost region, is the Q-M848 sub-clade with the most marked growth. Its distribution supports a Mexican origin as previously suggested [37], and a differentiation while moving southward. Structure-wise, no major changes were observed in the phylogeny of Q-M925. Notably, the presence of ancient samples from Meso and South America (I17889, 2.3 kya; PS_07, 1.5 kya; B_3) belonging to Q-Y12421 and Q-CTS748 suggests a territorial continuity of these lineages. Similarities can be recognised between the distribution of Q-M925 and Q-Z780, in that they both harbour a major, structured Mexican clade and a minor Isthmo-Colombian-specific lineage (Q-CTS748 and Q-Y12421 for Q-M925, Q-YP921 and Q-SA02 for Q-Z780, respectively). However, the spread of Q-M925 seems to have stopped with the entry in Colombia, as other Q-M848 sub-lineages characterise samples from South America.

In addition, we describe for the first time two geographically well-structured clades, Q-BY104773 and Q-BY15730, which provide new insights into the peopling of South America. They include samples from both the Andean mountain range and the Caribbean, thus adding a missing tile to the genetic history of the subcontinent. Indeed, Q-BY104773 (14.3 kya), which is represented by modern individuals from North-West Amazonia, is associated to a population growth slowly starting after 12.5 kya and then accelerating before 5 kya, around the time when signs of intensive agriculture appeared along the Neotropics [85]. A single ancient sample (A460; 5.1 kya) from Chilean Patagonia, classified as Q-BY104773*, would suggest the presence of the lineage amongst groups settled along the Pacific Coast, although its expansion was clearly unsuccessful outside of North-West Amazonia. The distinction between the Ayayema sample and the South American Q-BY104773 cluster would be in line with genomic evidence that consider this individual closer to the Lagoa Santa populations (10.4–9.8 kya) rather than to the rest of the Southern continent [11]. It is likely that Q-BY104773 was present amongst the first settlers coming from the Pacific Coast/Andes into the north-western Amazon (first signs of occupation in the area date back to 14–12 kya [86]) and that the continuous population growth detected represents their slow adaptation to the Amazonian environment. As previously pointed out [87], resources were consistent along the Pacific Coast, favouring a rapid migration, whereas tropical forests were not habitats with abundant and stable wild resources for hunters and gatherers, and it would have taken several generations for the early settlers to adjust to the new environment.

One Q-BY104773 sub-lineage, Q-BY139813, was also observed in ancient samples (~1.3–0.5 kya) from the lower Caribbean. Interestingly, these specimens were all excavated from Ceramic-associated sites, whereas ancient samples from archaeological contexts associated to the Archaic period (prior to the Ceramic) did not belong to the Q-BY104773 clade. This distribution provides further evidence for the migration of Ceramic Age people into the Lesser Antilles and northwards, starting from ~2 kya, and points at the north-western region of South America as their place of origin, in accordance with genomic data [42,43]. Moreover, the absence of Q-BY104773 in Cuba is in agreement with archaeological data suggesting a stop of the Ceramic migration at the Greater Antilles, probably due to the presence of Archaic-related groups [88]. In line with genomic analyses that detected “two distinct ancestries in Cuba around 2700 to 2500 calBP [...] before the arrival of Ceramic Age groups” [42], the two most ancient Cuban individuals (CIP009 and GUY002) were classified as Q-M848 and Q-Z780, respectively. It seems natural to suppose that the migration of Ceramic groups crossed Venezuela, but the absence of modern individuals from this country does not allow us to assert it confidently. Furthermore, Q-BY104773 was not observed in Ceramic-related samples from Venezuela, who instead appeared more related to Chibchan-speaking groups rather than to the Caribbean specimens, as also suggested by autosomal data [43]. Indeed, one 2.3 ky-old Venezuelan individual fell into the Isthmo-Colombian Q-Y12421. A further link between the Caribbean and the inner region of South America is attested by the minor clade Q-SK1965 (Figure S3), which collects modern individuals from Colombia, northern Brazil, and Puerto Rico.

Q-BY15730, the second major sub-clade reported here for the first time, tells a story similar to that of Q-BY104773. Q-BY15730, observed in modern individuals from North-West Amazonia, was dated at 13.2 kya, slightly before the first human traces in the area, and its phylogeography draws a displacement from Ecuador/Colombia into the Amazon region following river streams. The importance of rivers in the settlement and human activities in this part of South America was previously discussed by Arias et al. [89], and the distribution of Q-BY15730 points to a Pacific Coastal origin. It should be noted that Q-BY104773, which is of similar age and occupies roughly the same area as Q-BY15730, does not show such a marked cline in Colombia. A possible explanation for the peculiar distribution of Q-BY15730 could be that it is mainly found in Tukanoan groups, where the custom of patrilocality is particularly strong, thus strengthening the geographic separation of male lineages [36,89].

Moving along the Pacific Coast, three main lineages (Q-Z5906, Q-Z5908, and Q-Y780) are predominant. All three sub-clades stem directly from Q-M848 and are rather ancient, with estimated ages of 12.4 kya, 10.2 kya, and 9.4 kya, respectively. Their near complete absence outside the Andes attests the effectiveness of the mountain range in limiting the movement of people and is in agreement with genomic data reporting a clear separation between Andean and Amazonian populations [14,51,54,90]. Out of the three sub-clades, Q-Y780 contains a limited number of mostly LC individuals, which restricts its analysis, whereas Q-Z5906 and Q-Z5908 are comparable in distribution and level of structure. The Bayesian Skyline analysis of these last two sub-clades reveals an early population growth of Q-Z5908, starting from ~6.5 kya, shortly after the first appearance of crops in Peru [85], and the expansion of Q-Z5906, accompanied by a steady minor growth of Q-Z5908, from ~2.5 kya, in conjunction with the expansion of agriculture and the rise of sedentary societies in the region [91,92,93,94,95,96,97,98].

The remaining Q-M848 chromosomes either belong to small clusters or are ancestral for the markers defining the sub-lineages noted above. Although it is difficult to draw conclusions on the origin or dispersal of the minor sub-lineages, at the same time the observation of still undefined Q-M848 individuals hints at the existence of different, yet-to-be-identified Y-chromosome clades.

These results can be explained by the arrival of Q-Z780 and Q-M848 in Mexico prior to 15 kya, where they began differentiating while moving further South. This is in accordance with the scenario originally proposed by Sandoval et al. [99] and later by Battaglia et al. [79]. In addition, our results suggest that as the Pleistocene populations spread rapidly across the Southern continent, small groups settled in separate regions, differentiating locally. The age estimates of the most ancient, major, and minor Q-M848 sub-lineages would place these events around 14–12 kya (Table S2). With the subsequent adaptation to the new environmental niches, the scattered populations grew at different times (as shown in Figure 11), increasing the frequency of new local variants. Such a scenario is in agreement with the most ancient archaeological sites distributed across South America, attesting a stable occupation of the sub-continent around 14 kya [100].

As for the migration routes, the one along the Pacific Coast is evident in the distribution of the major Andean Q-Z5906 and Q-Z5908 lineages, whereas the newly reported Q-BY104773 and Q-BY15730 mark the entrance into the continental interior. The limited number of individuals sampled from the remaining regions of South America prevents us from fully comprehending times and modes of colonisation at the continental level. To interpret the high level of variability already visible in the few individuals collected from those areas would require further sampling along the Atlantic Coast as well as across the central part of South America.

## 5. Conclusions

Our analysis confirms haplogroups C-MPB373, C-P39, Q-Z780, Q-M848, and Q-Y4276 as the main Native American founding lineages. Moreover, the novel integration of modern and ancient samples allowed us to trace unsuccessful or extinct Y-chromosome lineages, revealing a broader haplogroup diversity among the human groups that initially entered the Americas that cannot be retrieved using present-day DNA data alone. Namely, C-L1373* was represented by the Brazilian 9.85 ky-old CP19, Q-YP4010* was found in the 1.8 ky-old Lovelock4 from Nevada, and pre-Q-F1096 was observed in the Tsimshian sample from South Alaska.

Q-Z780 and Q-M848, which include the majority of the American Indigenous Y chromosomes, were most likely carried by the first settlers of the continent, together with C-L1373. The distribution and age estimates of Q-Z780 and Q-M848 sub-lineages suggest that they reached Mexico before 15 kya. From there, whereas some Pleistocene populations started settling in the area giving rise to large local clusters (Q-YP921 and Q-CTS748, respectively), others proceeded southward, settling as small groups along the way, differentiating into local clades, like the Isthmian Q-SA02 and Q-Y12421, and the Andean Q-Z5906 and Q-Z5908. The last two, as well as Q-Z780, best delineate the Pacific coastal route, where consistently favourable conditions allowed a rapid spread.

Thanks to the identification of the two novel Q-M848 sub-branches, Q-BY104773 and Q-BY15730, we provide the first Y-chromosome evidence of South American inland peopling. In particular, Q-BY15730 (13.2 kya) marks an early migration from Ecuador/Colombia into the Amazonian area, in accordance with traces of human settlement in the region dating back to 12 kya. Similarly, Q-BY104773 (14.3 kya) probably reached the interior of Colombia from the Pacific Coast and differentiated in North-West Amazonia. Moreover, the Q-BY104773 sub-lineage Q-BY139813 provides further evidence for the migration of Ceramic Age groups from the northern region of South America into the Lesser Antilles.

All the Q-M848 sub-clades noted above underwent local population expansions once adapted to specific environmental niches, concomitantly with the improvement of climatic and subsistence conditions.

In conclusion, this work assembles the most comprehensive Native American Y-chromosome dataset to date and greatly improves the phylogenetic resolution of haplogroup Q. Furthermore, it helps to shed light on some aspects of the peopling of the double continent, especially in South America, and emphasises that additional sampling in areas that are still underrepresented, both for modern and ancient individuals, is necessary to fully reconstruct the genetic history and demography of Indigenous Americans.

## Data Availability

Not applicable.

## Supplementary Materials

The following supporting information can be downloaded at: https://www.mdpi.com/article/10.3390/genes13020220/s1, Figure S1: Geographic localisation of ancient and modern American samples included in the phylogenetic analysis. Ancient individuals are indicated by their median calibrated age (cal BP, see Table S1 for details), or by a cross when the age was not available; Figure S2: Phylogenetic tree of all the samples included in the study (Table S1). Ancient individuals are indicated by a cross; Figure S3: Phylogeography of the C-L1373 branches discussed in the text. Ancient individuals are indicated by their median calibrated age (cal BP, see Supplementary Table S1 for details). The placement of the North American lineage C-P39 (dashed line in red) is inferred from [38]; Figure S4: Phylogeny and phylogeography of the minor Q-M848 sub-lineages. In the phylogeny, the estimated ages of the nodes (±StDev) are reported in kya; Table S1: Complete dataset; Table S2: Age estimates of haplogroup C and Q branches, compared to those previously provided by [37,38].

## Code Availability

Scripts for the conversion of multi-VCFs to multi-FASTA file useful as input for Beast analyses can be found at this link (https://github.com/raveancic/fromhaplomulti-VCF2FASTA, accessed on 21 January 2022).

## References

[1] Greenberg J.H., Turner C.G., Zegura S.L., Campbell L., Fox J.A., Laughlin W.S., Weiss K.M., Woolford E. (1986). The Settlement of the Americas: A comparison of the linguistic, dental, and genetic evidence. Curr. Anthropol..

[2] Torroni A., Schurr T.G., Cabell M.F., Brown M.D., Neel J.V., Larsen M., Smith D.G., Vullo C.M., Wallace D.C. (1993). Asian affinities and continental radiation of the four founding Native American mtDNAs. Am. J. Hum. Genet..

[3] Torroni A., Sukernik R.I., Schurr T.G., Starikorskaya Y.B., Cabell M.F., Crawford M.H., Comuzzie A.G., Wallace D.C. (1993). MtDNA variation of aboriginal Siberians reveals distinct genetic affinities with Native Americans. Am. J. Hum. Genet..

[4] Pena S.D.J., Santos F.R., Bianchi N.O., Bravi C.M., Carnese F.R., Rothhammer F., Gerelsaikhan T., Munkhtuja B., Oyunsuren T. (1995). A major founder Y–chromosome haplotype in Amerindians. Nat. Genet..

[5] Dillehay T.D. (2000). A New Prehistory: Settlement of the Americas.

[6] Lahaye C., Hernandez M., Boëda E., Felice G.D., Guidon N., Hoeltz S., Lourdeau A., Pagli M., Pessis A.-M., Rasse M. (2013). Human occupation in South America by 20,000 BC: The Toca Da Tira Peia site, Piauí, Brazil. J. Archaeol. Sci..

[7] Ardelean C.F., Becerra-Valdivia L., Pedersen M.W., Schwenninger J.-L., Oviatt C.G., Macías-Quintero J.I., Arroyo-Cabrales J., Sikora M., Ocampo-Díaz Y.Z.E., Rubio-Cisneros I.I. (2020). Evidence of human occupation in Mexico around the Last Glacial Maximum. Nature.

[8] Dillehay T.D., Ocampo C., Saavedra J., Sawakuchi A.O., Vega R.M., Pino M., Collins M.B., Cummings L.S., Arregui I., Villagran X.S. (2015). New archaeological evidence for an early human presence at Monte Verde, Chile. PLoS ONE.

[9] Braje T.J., Dillehay T.D., Erlandson J.M., Klein R.G., Rick T.C. (2017). Finding the first Americans. Science.

[10] Raghavan M., Steinrücken M., Harris K., Schiffels S., Rasmussen S., DeGiorgio M., Albrechtsen A., Valdiosera C., Ávila-Arcos M.C., Malaspinas A.-S. (2015). Genomic evidence for the Pleistocene and recent population history of Native Americans. Science.

[11] Moreno-Mayar J.V., Vinner L., de Barros Damgaard P., de la Fuente C., Chan J., Spence J.P., Allentoft M.E., Vimala T., Racimo F., Pinotti T. (2018). Early human dspersals within the Americas. Science.

[12] Scheib C.L., Li H., Desai T., Link V., Kendall C., Dewar G., Griffith P.W., Mörseburg A., Johnson J.R., Potter A. (2018). Ancient human parallel lineages within North America contributed to a coastal expansion. Science.

[13] Achilli A., Olivieri A., Semino O., Torroni A. (2018). Ancient human genomes—keys to understanding our past. Science.

[14] Posth C., Nakatsuka N., Lazaridis I., Skoglund P., Mallick S., Lamnidis T.C., Rohland N., Nägele K., Adamski N., Bertolini E. (2018). Reconstructing the deep population history of Central and South America. Cell.

[15] Capodiferro M.R., Aram B., Raveane A., Rambaldi Migliore N., Colombo G., Ongaro L., Rivera J., Mendizábal T., Hernández-Mora I., Tribaldos M. (2021). Archaeogenomic distinctiveness of the Isthmo-Colombian area. Cell.

[16] Bonilla C., Bertoni B., González S., Cardoso H., Brum-Zorrilla N., Sans M. (2004). Substantial Native American female contribution to the population of Tacuarembó, Uruguay, reveals past episodes of sex-biased gene flow. Am. J. Hum. Biol..

[17] Bolnick D.A., Bolnick D.I., Smith D.G. (2006). Asymmetric male and female genetic histories among Native Americans from eastern North America. Mol. Biol. Evol..

[18] Grugni V., Battaglia V., Perego U.A., Raveane A., Lancioni H., Olivieri A., Ferretti L., Woodward S.R., Pascale J.M., Cooke R. (2015). Exploring the Y chromosomal ancestry of modern Panamanians. PLoS ONE.

[19] Rambaldi Migliore N., Colombo G., Capodiferro M.R., Mazzocchi L., Chero Osorio A.M., Raveane A., Tribaldos M., Perego U.A., Mendizábal T., Montón A.G. (2021). Weaving mitochondrial DNA and Y-chromosome variation in the Panamanian genetic canvas. Genes.

[20] Bryc K., Durand E.Y., Macpherson J.M., Reich D., Mountain J.L. (2015). The genetic ancestry of African Americans, Latinos, and European Americans across the United States. Am. J. Hum. Genet..

[21] Ongaro L., Scliar M.O., Flores R., Raveane A., Marnetto D., Sarno S., Gnecchi-Ruscone G.A., Alarcón-Riquelme M.E., Patin E., Wangkumhang P. (2019). The genomic impact of European colonization of the Americas. Curr. Biol..

[22] Lind J.M., Hutcheson-Dilks H.B., Williams S.M., Moore J.H., Essex M., Ruiz-Pesini E., Wallace D.C., Tishkoff S.A., O’Brien S.J., Smith M.W. (2006). Elevated male European and female African contributions to the genomes of African American individuals. Hum. Genet..

[23] Bryc K., Auton A., Nelson M.R., Oksenberg J.R., Hauser S.L., Williams S., Froment A., Bodo J.-M., Wambebe C., Tishkoff S.A. (2010). Genome-wide patterns of population structure and admixture in West Africans and African Americans. Proc. Natl. Acad. Sci. USA.

[24] Bryc K., Velez C., Karafet T., Moreno-Estrada A., Reynolds A., Auton A., Hammer M., Bustamante C.D., Ostrer H. (2010). Genome-wide patterns of population structure and admixture among Hispanic/Latino Populations. Proc. Natl. Acad. Sci. USA.

[25] Cox M.P., Karafet T.M., Lansing J.S., Sudoyo H., Hammer M.F. (2010). Autosomal and X-linked single nucleotide polymorphisms reveal a steep Asian–Melanesian ancestry cline in eastern Indonesia and a sex bias in admixture rates. Proc. Royal Soc. B.

[26] Verdu P., Becker N.S.A., Froment A., Georges M., Grugni V., Quintana-Murci L., Hombert J.-M., Van der Veen L., Le Bomin S., Bahuchet S. (2013). Sociocultural behavior, sex-biased admixture, and effective population sizes in central African Pygmies and Non-Pygmies. Mol. Biol. Evol..

[27] Ongaro L., Molinaro L., Flores R., Marnetto D., Capodiferro M.R., Alarcón-Riquelme M.E., Moreno-Estrada A., Mabunda N., Ventura M., Tambets K. (2021). Evaluating the impact of sex-biased genetic admixture in the Americas through the analysis of haplotype data. Genes.

[28] Soares P., Ermini L., Thomson N., Mormina M., Rito T., Röhl A., Salas A., Oppenheimer S., Macaulay V., Richards M.B. (2009). Correcting for purifying selection: An improved human mitochondrial molecular clock. Am. J. Hum. Genet..

[29] Karmin M., Saag L., Vicente M., Sayres M.A.W., Järve M., Talas U.G., Rootsi S., Ilumäe A.-M., Mägi R., Mitt M. (2015). A recent bottleneck of Y chromosome diversity coincides with a global change in culture. Genome Res..

[30] Balanovsky O. (2017). Toward a consensus on SNP and STR mutation rates on the human Y-chromosome. Hum. Genet..

[31] Achilli A., Perego U.A., Bravi C.M., Coble M.D., Kong Q.-P., Woodward S.R., Salas A., Torroni A., Bandelt H.-J. (2008). The phylogeny of the four pan-American mtDNA haplogroups: Implications for evolutionary and disease studies. PLoS ONE.

[32] Perego U.A., Achilli A., Angerhofer N., Accetturo M., Pala M., Olivieri A., Kashani B.H., Ritchie K.H., Scozzari R., Kong Q.-P. (2009). Distinctive Paleo-Indian migration routes from Beringia marked by two rare MtDNA haplogroups. Curr. Biol..

[33] Perego U.A., Angerhofer N., Pala M., Olivieri A., Lancioni H., Kashani B.H., Carossa V., Ekins J.E., Gómez-Carballa A., Huber G. (2010). The initial peopling of the Americas: A growing number of founding mitochondrial genomes from Beringia. Genome Res..

[34] Brandini S., Bergamaschi P., Cerna M.F., Gandini F., Bastaroli F., Bertolini E., Cereda C., Ferretti L., Gómez-Carballa A., Battaglia V. (2018). The Paleo-Indian entry into South America according to mitogenomes. Mol. Biol. Evol..

[35] Poznik G.D., Henn B.M., Yee M.-C., Sliwerska E., Euskirchen G.M., Lin A.A., Snyder M., Quintana-Murci L., Kidd J.M., Underhill P.A. (2013). Sequencing Y chromosomes resolves discrepancy in time to common ancestor of males versus females. Science.

[36] Arias L., Schröder R., Hübner A., Barreto G., Stoneking M., Pakendorf B. (2018). Cultural innovations influence patterns of genetic diversity in Northwestern Amazonia. Mol. Biol. Evol..

[37] Grugni V., Raveane A., Ongaro L., Battaglia V., Trombetta B., Colombo G., Capodiferro M.R., Olivieri A., Achilli A., Perego U.A. (2019). Analysis of the human Y-chromosome haplogroup Q characterizes ancient population movements in Eurasia and the Americas. BMC Biol..

[38] Pinotti T., Bergström A., Geppert M., Bawn M., Ohasi D., Shi W., Lacerda D.R., Solli A., Norstedt J., Reed K. (2019). Y chromosome sequences reveal a short Beringian standstill, rapid expansion, and early population structure of Native American Founders. Curr. Biol..

[39] (2015). The 1000 Genomes Project Consortium. Nature.

[40] Mallick S., Li H., Lipson M., Mathieson I., Gymrek M., Racimo F., Zhao M., Chennagiri N., Nordenfelt S., Tandon A. (2016). The Simons Genome Diversity Project: 300 genomes from 142 diverse populations. Nature.

[41] Bergström A., McCarthy S.A., Hui R., Almarri M.A., Ayub Q., Danecek P., Chen Y., Felkel S., Hallast P., Kamm J. (2020). Insights into human genetic variation and population history from 929 diverse genomes. Science.

[42] Nägele K., Posth C., Iraeta Orbegozo M., Chinique de Armas Y., Hernández Godoy S.T., González Herrera U.M., Nieves-Colón M.A., Sandoval-Velasco M., Mylopotamitaki D., Radzeviciute R. (2020). Genomic insights into the early peopling of the Caribbean. Science.

[43] Fernandes D.M., Sirak K.A., Ringbauer H., Sedig J., Rohland N., Cheronet O., Mah M., Mallick S., Olalde I., Culleton B.J. (2021). A genetic history of the pre-contact Caribbean. Nature.

[44] Chambers J.C., Abbott J., Zhang W., Turro E., Scott W.R., Tan S.-T., Afzal U., Afaq S., Loh M., Lehne B. (2014). The South Asian genome. PLoS ONE.

[45] Prüfer K., Racimo F., Patterson N., Jay F., Sankararaman S., Sawyer S., Heinze A., Renaud G., Sudmant P.H., de Filippo C. (2014). The complete genome sequence of a Neanderthal from the Altai Mountains. Nature.

[46] Rasmussen M., Li Y., Lindgreen S., Pedersen J.S., Albrechtsen A., Moltke I., Metspalu M., Metspalu E., Kivisild T., Gupta R. (2010). Ancient human genome sequence of an extinct Palaeo-Eskimo. Nature.

[47] Rasmussen M., Anzick S.L., Waters M.R., Skoglund P., DeGiorgio M., Stafford T.W., Rasmussen S., Moltke I., Albrechtsen A., Doyle S.M. (2014). The genome of a late Pleistocene human from a Clovis burial site in Western Montana. Nature.

[48] Rasmussen M., Sikora M., Albrechtsen A., Korneliussen T.S., Moreno-Mayar J.V., Poznik G.D., Zollikofer C.P.E., Ponce de León M.S., Allentoft M.E., Moltke I. (2015). The ancestry and affiliations of Kennewick Man. Nature.

[49] Malaspinas A.-S., Lao O., Schroeder H., Rasmussen M., Raghavan M., Moltke I., Campos P.F., Sagredo F.S., Rasmussen S., Gonçalves V.F. (2014). Two ancient human genomes reveal Polynesian ancestry among the Indigenous Botocudos of Brazil. Curr. Biol..

[50] de la Fuente C., Ávila-Arcos M.C., Galimany J., Carpenter M.L., Homburger J.R., Blanco A., Contreras P., Dávalos D.C., Reyes O., Roman M.S. (2018). Genomic insights into the origin and diversification of late maritime hunter-gatherers from the Chilean Patagonia. Proc. Natl. Acad. Sci. USA.

[51] Lindo J., Haas R., Hofman C., Apata M., Moraga M., Verdugo R.A., Watson J.T., Viviano Llave C., Witonsky D., Beall C. (2018). The genetic prehistory of the Andean Highlands 7000 years BP though European contact. Sci. Adv..

[52] Flegontov P., Altınışık N.E., Changmai P., Rohland N., Mallick S., Adamski N., Bolnick D.A., Broomandkhoshbacht N., Candilio F., Culleton B.J. (2019). Palaeo-Eskimo genetic ancestry and the peopling of Chukotka and North America. Nature.

[53] Sikora M., Pitulko V.V., Sousa V.C., Allentoft M.E., Vinner L., Rasmussen S., Margaryan A., de Barros Damgaard P., de la Fuente C., Renaud G. (2019). The population history of Northeastern Siberia since the Pleistocene. Nature.

[54] Nakatsuka N., Lazaridis I., Barbieri C., Skoglund P., Rohland N., Mallick S., Posth C., Harkins-Kinkaid K., Ferry M., Harney É. (2020). A paleogenomic reconstruction of the deep population history of the Andes. Cell.

[55] Nakatsuka N., Luisi P., Motti J.M.B., Salemme M., Santiago F., D’Angelo del Campo M.D., Vecchi R.J., Espinosa-Parrilla Y., Prieto A., Adamski N. (2020). Ancient genomes in South Patagonia reveal population movements associated with technological shifts and geography. Nat. Commun..

[56] Li H., Durbin R. (2009). Fast and accurate short read alignment with Burrows–Wheeler transform. Bioinformatics.

[57] Garrison E., Marth G. (2012). Haplotype-based variant detection from short-read sequencing. arXiv.

[58] Chen H., Lu Y., Lu D., Xu S. (2021). Y-LineageTracker: A high-throughput analysis framework for Y-chromosomal next-generation sequencing data. BMC Bioinform..

[59] Ortiz E.M. Vcf2phylip v2.0: Convert a VCF matrix into several matrix formats for phylogenetic analysis. Software 2019. https://zenodo.org/record/2540861#.Ye-evTgzaUk.

[60] Stamatakis A. (2014). RAxML version 8: A tool for phylogenetic analysis and post-analysis of large phylogenies. Bioinformatics.

[61] Huerta-Cepas J., Serra F., Bork P. (2016). ETE3: Reconstruction, analysis, and visualization of phylogenomic data. Mol. Biol. Evol..

[62] Martiniano R., De Sanctis B., Hallast P., Durbin R. (2020). Placing ancient DNA sequences into reference phylogenies. bioRxiv.

[63] Miles A., pyup io bot, Murillo R., Ralph P., Harding N., Pisupati R., Rae S., Millar T. Cggh/Scikit-Allel: V1.3.3. 2021. https://zenodo.org/record/4759368#.Ye-e-jgzaUk.

[64] Bouckaert R., Vaughan T.G., Barido-Sottani J., Duchêne S., Fourment M., Gavryushkina A., Heled J., Jones G., Kühnert D., Maio N.D. (2019). BEAST 2.5: An advanced software platform for Bayesian evolutionary analysis. PLoS Comput. Biol..

[65] Kumar S., Stecher G., Li M., Knyaz C., Tamura K. (2018). MEGA X: Molecular evolutionary genetics analysis across computing platforms. Mol. Biol. Evol..

[66] Rambaut A., Drummond A.J., Xie D., Baele G., Suchard M.A. (2018). Posterior summarization in Bayesian phylogenetics using Tracer 1.7. Syst. Biol..

[67] R Core Team R (2021). A Language and Environment for Statistical Computing.

[68] Karafet T.M., Zegura S.L., Posukh O., Osipova L., Bergen A., Long J., Goldman D., Klitz W., Harihara S., de Knijff P. (1999). Ancestral Asian source(s) of New World Y-chromosome founder haplotypes. Am. J. Hum. Genet..

[69] Zegura S.L., Karafet T.M., Zhivotovsky L.A., Hammer M.F. (2004). High-Resolution SNPs and Microsatellite Haplotypes Point to a Single, Recent entry of Native American Y chromosomes into the Americas. Mol. Biol. Evol..

[70] Malhi R.S., Gonzalez-Oliver A., Schroeder K.B., Kemp B.M., Greenberg J.A., Dobrowski S.Z., Smith D.G., Resendez A., Karafet T., Hammer M. (2008). Distribution of Y chromosomes among Native North Americans: A study of Athapaskan population history. Am. J. Phys. Anthropol..

[71] Geppert M., Baeta M., Núñez C., Martínez-Jarreta B., Zweynert S., Cruz O.W.V., González-Andrade F., González-Solorzano J., Nagy M., Roewer L. (2011). Hierarchical Y-SNP assay to study the hidden diversity and phylogenetic relationship of Native populations in South America. Forensic Sci. Int. Genet..

[72] Roewer L., Nothnagel M., Gusmão L., Gomes V., González M., Corach D., Sala A., Alechine E., Palha T., Santos N. (2013). Continent-wide decoupling of Y-chromosomal genetic variation from language and geography in Native South Americans. PLoS Genet..

[73] Mezzavilla M., Geppert M., Tyler-Smith C., Roewer L., Xue Y. (2015). Insights into the origin of rare haplogroup C3* Y chromosomes in South America from high-density autosomal SNP genotyping. Forensic Sci. Int. Genet..

[74] Zhong H., Shi H., Qi X.-B., Duan Z.-Y., Tan P.-P., Jin L., Su B., Ma R.Z. (2011). Extended Y chromosome investigation suggests postglacial migrations of modern humans into East Asia via the northern route. Mol. Biol. Evol..

[75] Wei L.-H., Wang L.-X., Wen S.-Q., Yan S., Canada R., Gurianov V., Huang Y.-Z., Mallick S., Biondo A., O’Leary A. (2018). Paternal origin of Paleo-Indians in Siberia: Insights from Y-chromosome sequences. Eur. J. Hum. Genet..

[76] Chiaroni J., Underhill P.A., Cavalli-Sforza L.L. (2009). Y chromosome diversity, human expansion, drift, and cultural evolution. Proc. Natl. Acad. Sci. USA.

[77] Huang Y.-Z., Pamjav H., Flegontov P., Stenzl V., Wen S.-Q., Tong X.-Z., Wang C.-C., Wang L.-X., Wei L.-H., Gao J.-Y. (2018). Dispersals of the Siberian Y-chromosome haplogroup Q in Eurasia. Mol. Genet. Genom..

[78] Jota M.S., Lacerda D.R., Sandoval J.R., Vieira P.P.R., Ohasi D., Santos-Júnior J.E., Acosta O., Cuellar C., Revollo S., Paz-y-Miño C. (2016). New Native South American Y chromosome lineages. J. Hum. Genet..

[79] Battaglia V., Grugni V., Perego U.A., Angerhofer N., Gomez-Palmieri J.E., Woodward S.R., Achilli A., Myres N., Torroni A., Semino O. (2013). The first peopling of South America: New evidence from Y-chromosome haplogroup Q. PLoS ONE.

[80] Montinaro F., Busby G.B.J., Pascali V.L., Myers S., Hellenthal G., Capelli C. (2015). Unravelling the hidden ancestry of American admixed populations. Nat. Commun..

[81] Fortes-Lima C., Gessain A., Ruiz-Linares A., Bortolini M.-C., Migot-Nabias F., Bellis G., Moreno-Mayar J.V., Restrepo B.N., Rojas W., Avendaño-Tamayo E. (2017). Genome-wide ancestry and demographic history of African-descendant Maroon communities from French Guiana and Suriname. Am. J. Hum. Genet..

[82] Chacón-Duque J.-C., Adhikari K., Fuentes-Guajardo M., Mendoza-Revilla J., Acuña-Alonzo V., Barquera R., Quinto-Sánchez M., Gómez-Valdés J., Everardo Martínez P., Villamil-Ramírez H. (2018). Latin Americans show wide-spread Converso ancestry and imprint of local Native ancestry on physical appearance. Nat. Commun..

[83] Willerslev E., Meltzer D.J. (2021). Peopling of the Americas as inferred from ancient genomics. Nature.

[84] Martiniano R., Garrison E., Jones E.R., Manica A., Durbin R. (2020). Removing reference bias and improving indel calling in ancient DNA data analysis by mapping to a sequence variation graph. Genome Biol..

[85] Piperno D.R. (2011). The origins of plant cultivation and domestication in the New World tropics: Patterns, process, and new developments. Curr. Anthropol..

[86] Morcote-Ríos G., Aceituno F.J., Iriarte J., Robinson M., Chaparro-Cárdenas J.L. (2021). Colonisation and early peopling of the Colombian Amazon during the late Pleistocene and the early Holocene: New evidence from La Serranía La Lindosa. Quat. Int..

[87] Piperno D.R., Pearsall D.M. (1998). The Origins of Agriculture in the Lowland Neotropics.

[88] Keegan W.F. (2000). West Indian archaeology. 3. Ceramic age. J. Archaeol. Res..

[89] Arias L., Barbieri C., Barreto G., Stoneking M., Pakendorf B. (2018). High-resolution mitochondrial DNA analysis sheds light on human diversity, cultural interactions, and population mobility in Northwestern Amazonia. Am. J. Phys. Anthropol..

[90] Barbieri C., Barquera R., Arias L., Sandoval J.R., Acosta O., Zurita C., Aguilar-Campos A., Tito-Álvarez A.M., Serrano-Osuna R., Gray R.D. (2019). The current genomic landscape of Western South America: Andes, Amazonia, and Pacific Coast. Mol. Biol. Evol..

[91] Rick J.W. (1988). The Character and Context of Highland Preceramic Society. Peruvian Prehistory: An Overview of Pre-Inca and Inca Society.

[92] Rivera M.A. (1995). The preceramic Chinchorro mummy complex of northern Chile: Context, style, and purpose. Tombs for the Living: Andean Mortuary Practices.

[93] Dillehay T.D., Rossen J., Andres T.C., Williams D.E. (2007). Preceramic adoption of peanut, squash, and sotton in northern Peru. Science.

[94] Aldenderfer M.S., Silverman H., Isbell W.H. (2008). High elevation foraging societies. The Handbook of South American Archaeology.

[95] Arriaza B.T., Standen V.G., Cassman V., Santoro C.M., Silverman H., Isbell W.H. (2008). Chinchorro culture: Pioneers of the Coast of the Atacama Desert. The Handbook of South American Archaeology.

[96] Hastorf C.A., Silverman H., Isbell W.H. (2008). The formative period in the Titicaca Basin. The Handbook of South American Archaeology.

[97] Pozorski S., Pozorski T., Silverman H., Isbell W.H. (2008). Early cultural complexity on the Coast of Peru. The Handbook of South American Archaeology.

[98] Quilter J. (2013). The Ancient Central Andes.

[99] Sandoval K., Moreno-Estrada A., Mendizabal I., Underhill P.A., Lopez-Valenzuela M., Peñaloza-Espinosa R., Lopez-Lopez M., Buentello-Malo L., Avelino H., Calafell F. (2012). Y-chromosome diversity in Native Mexicans reveals continental transition of genetic structure in the Americas. Am. J. Phys. Anthropol..

[100] Waters M.R. (2019). Late Pleistocene exploration and settlement of the Americas by modern humans. Science.

