# Supplementary material for: Overview of the Americas’ First Peopling from a Patrilineal Perspective: New Evidence from the Southern Continent
Source: Genes (Basel). 2022 Jan 25;13(2):220. doi: 10.3390/genes13020220 (PMC8871784; doi:10.3390/genes13020220)
Supplement: Supplementary file 1 [file genes-13-00220-s001.zip › Supplementary_Figures_S1-S4.pdf]

Supplementary Figures

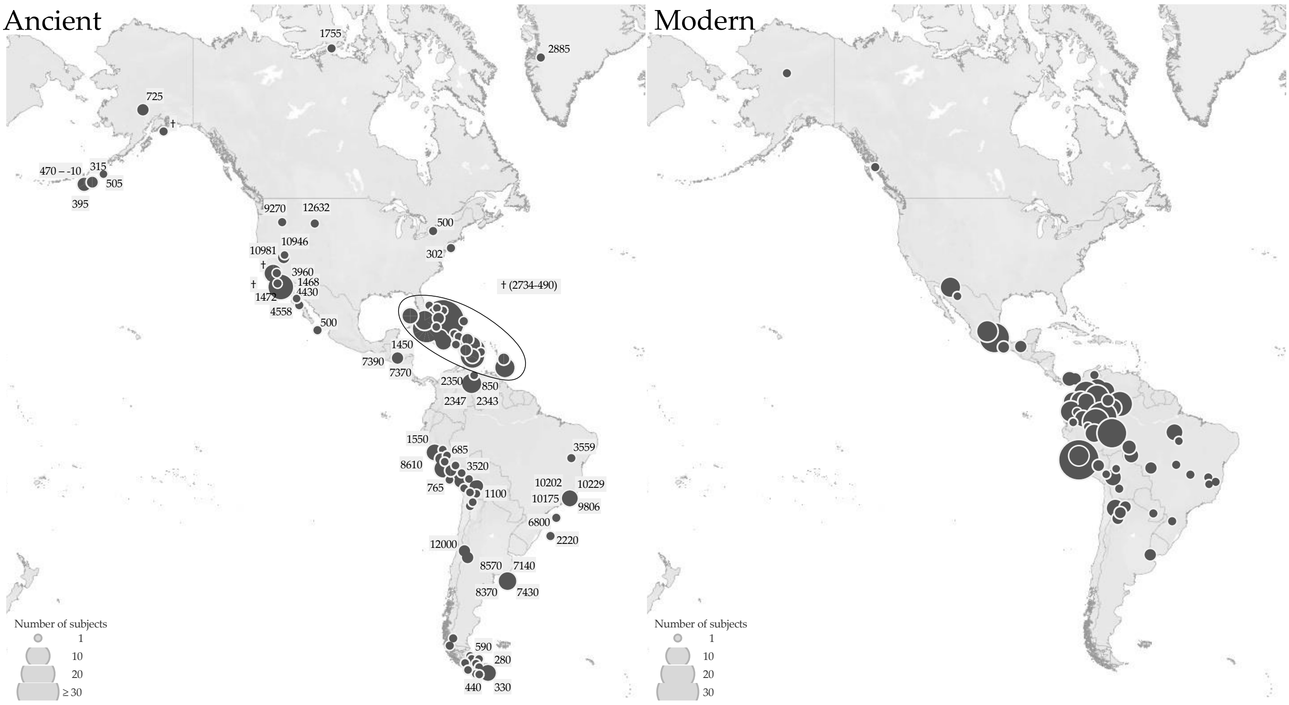

Figure S1. Geographic localisation of ancient and modern American samples included in the phylogenetic analysis. Ancient individuals are indicated by their median calibrated age (cal BP, see Supplementary Table S1 for details), or by a cross when the age was not available.

[Figure S2 is present as a separate file]

Figure S2. Phylogenetic tree of all the samples included in the study (Supplementary Table S1). Ancient individuals are indicated by a cross.

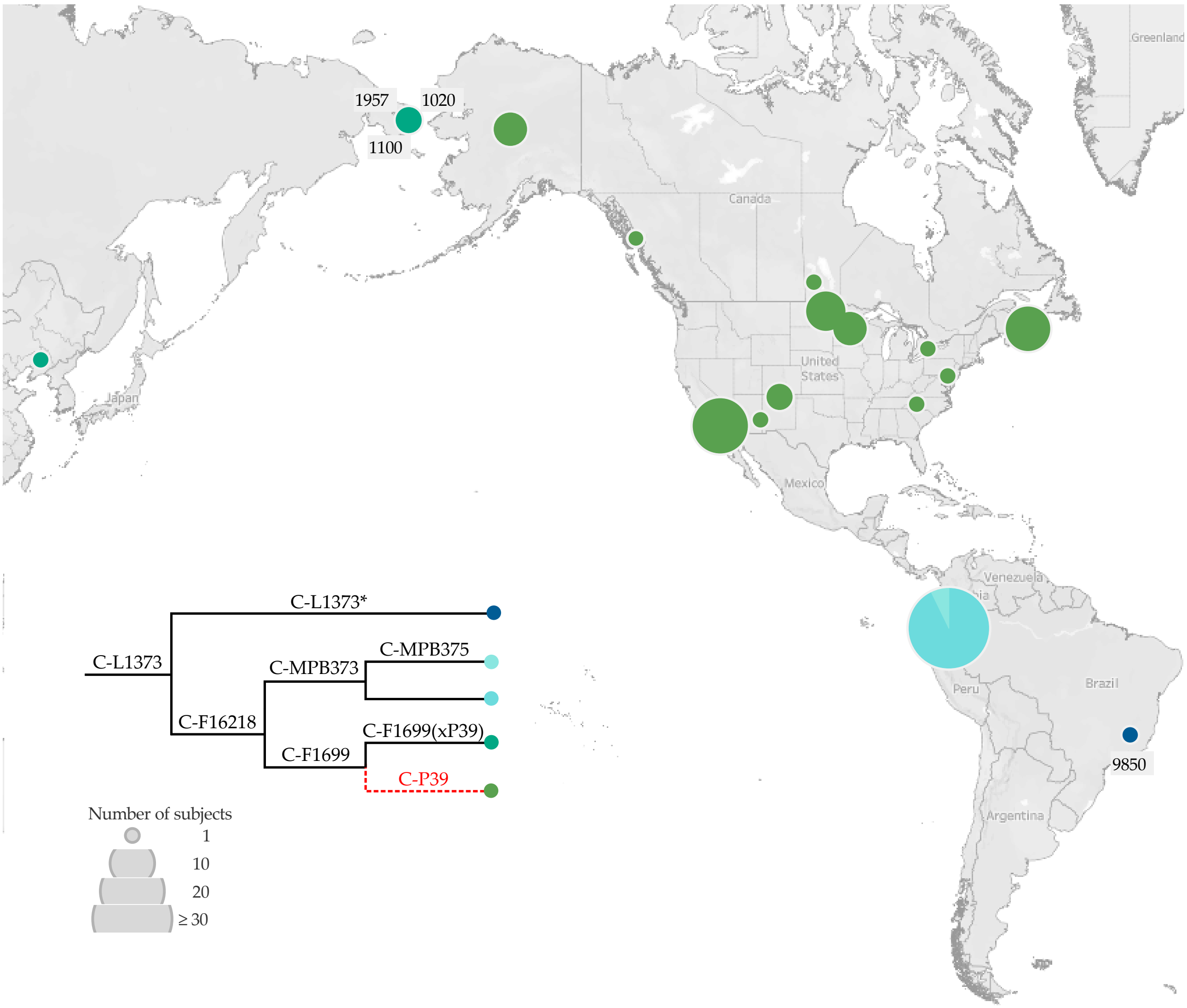

Figure S3. Phylogeography of the C-L1373 (ISOGG nomenclature: C2b - http://www.isogg.org/tree/ Date of access: 19 January 2022) branches discussed in the text. Ancient individuals are indicated by their median calibrated age (cal BP, see Supplementary Table S1 for details). The placement of the North American lineage C-P39 (dashed line in red) is inferred from Pinotti et al (2019).

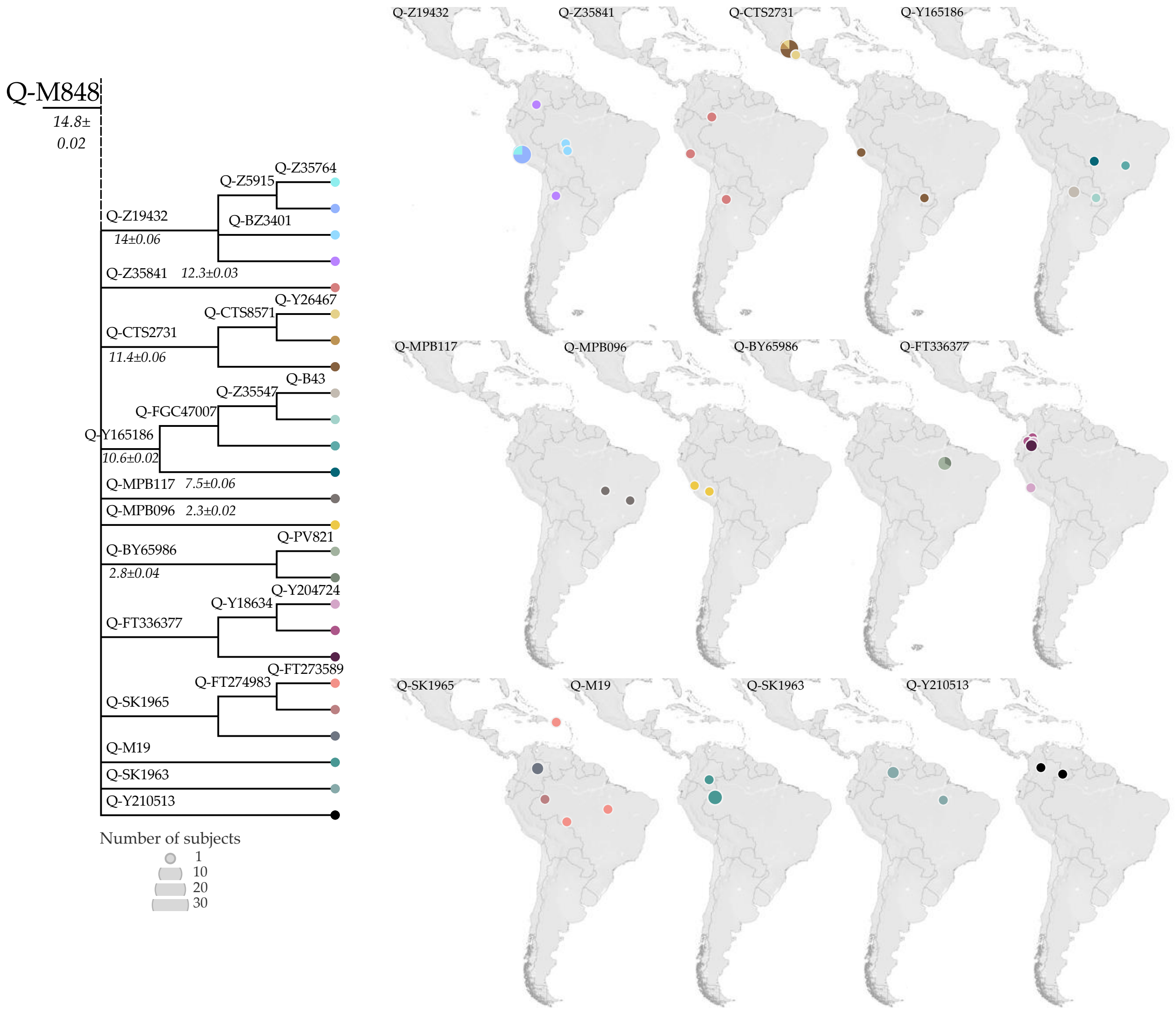

Figure S4. Phylogeny and phylogeography of the minor Q-M848 sub-lineages. In the phylogeny, the estimated ages of the nodes (± StDev) are reported in kya.
