# Supplementary material for: Overview of the Americas’ First Peopling from a Patrilineal Perspective: New Evidence from the Southern Continent
Source: Genes (Basel). 2022 Jan 25;13(2):220. doi: 10.3390/genes13020220 (PMC8871784; doi:10.3390/genes13020220)

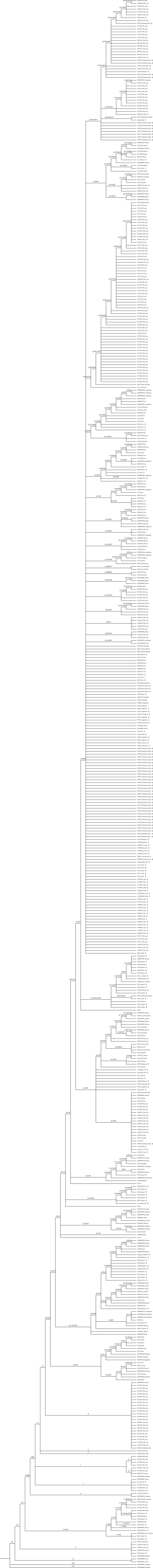

Supplement: Supplementary file 1 [file Supplementary_FigureS2.pdf › Supplementary_FigureS2.pdf]
